# Preoperative High Sleep Quality Predicts Further Decrease in Length of Stay after Total Joint Arthroplasty under Enhanced Recovery Short‐stay Program: Experience in 604 Patients from a Single Team

**DOI:** 10.1111/os.13382

**Published:** 2022-07-20

**Authors:** Zichuan Ding, Jinlong Li, Bing Xu, Jian Cao, Hao Li, Zongke Zhou

**Affiliations:** ^1^ Department of Orthopedics, West China Hospital/West China School of Medicine Sichuan University Chengdu P. R. China; ^2^ Department of Orthopedics Gansu Provincial Hospital Lanzhou P. R. China; ^3^ Department of Orthopedics Chengdu Second People's Hospital Chengdu P. R. China

**Keywords:** Enhanced recovery after surgery, Length of stay, Sleep quality, Total hip arthroplasty, Total knee arthroplasty

## Abstract

**Objective:**

To investigate the safety, efficiency and cost of total joint arthroplasty (TJA) under the enhanced recovery after surgery (ERAS) program and identify predictors facilitating further decrease in length of stay (LOS).

**Methods:**

We retrospectively collected the information of patients who underwent primary unilateral TJA by a single surgical team between January 2017 and June 2019. A total of 604 patients with LOS ≤ 3 was enrolled in this study. All patients completed 12‐month or longer follow‐up. Patients received the same ERAS protocol, mainly including preoperative preparation (patient education, preoperative functional exercises, nutritional support), blood management, pain management, sleep management, prevention of infection, prevention of thrombosis and strict discharge criteria. Preoperative characteristics of patients were collected from the medical record system and were compared between the LOS ≤ 2 group and the LOS = 3 group. Factors with significant difference were included in multivariate logistic regression analysis to find independent preoperative predictors for LOS. Joint function at the latest follow‐up, adverse events rate and hospitalization costs were compared between the LOS ≤ 2 group and the LOS = 3 group.

**Results:**

Of the enrolled 604 patients, 271 patients (44.9%) had a LOS of 2 days or less while 333 patients (55.1%) had a LOS of 3 days. Pittsburgh Sleep Quality Index score (odds ratio [OR] = 1.084, 95% confidence interval [CI] = 1.024–1.147, *P* = 0.005), preoperative albumin level (OR = 0.945, 95% CI = 0.905–0.988, *P* = 0.012), digestive diseases (OR = 1.084, 95% CI = 1.024–1.147, *P* = 0.005) and total hip arthroplasty (THA) (OR = 0.273, 95% CI = 0.170–0.439, *P* < 0.001) were predictors of LOS ≤ 2 in the multivariate logistic analysis model. The postoperative joint function scores and adverse event rates were comparable between the LOS ≤ 2 group and the LOS = 3 group. The hospital costs were lower in the LOS ≤ 2 group than the LOS = 3 group.

**Conclusion:**

Under the rigorous ERAS program, 2‐day discharge in unselected TJA patients can be routinely applied. Patients with high preoperative sleep quality, high preoperative albumin level, free of digestive disease and undergoing THA procedure are more likely to be discharged within 2 days.

## Graphical abstract

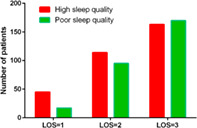

## Introduction

Total joint arthroplasty (TJA), including total hip arthroplasty (THA) and total knee arthroplasty (TKA), is considered to be the most successful procedure for end‐stage joint diseases in the lower limb. Since the population ages and more patients with high life quality expectancy choose to undergo TJA procedure, the number of TJAs was predicted to grow steadily.^1^ The financial burden has increased the pressures to reduce hospital length of stay (LOS) and patient's cost as the longer LOS was associated with high patient's cost. Great effort has been made by surgeons to decrease the postoperative LOS after TJA, but the ultimate goal of outpatient arthroplasty in unselected patients is still far from being realized.

With the main idea of reaching the optimal condition of the patients for the procedure through a series of perioperative managements, the enhanced recovery after surgery (ERAS) program is an emerging field in TJA surgery and has attracted great attention in recent years.^2^ The ERAS program advocates evidence‐based interventions to decrease surgical trauma and stress response by a multimodal approach. It promotes a series of perioperative managements that focus on rapid recovery, reducing postoperative complications, early discharge, reducing costs, and improving patient satisfaction. ERAS has been proved to contribute to decreasing LOS, reducing hospital costs, reducing complication and readmission rates, improving patient's satisfaction after TJA.^3^

The LOS after TJA was reported to reduce by a large margin without increasing related complication rates under the ERAS program.^4^, ^5^ Vendittoli *et al*. reported that LOS, an important outcome of ERAS program evaluation and the main factor of success of TJA, was significantly reduced after ERAS implementation.^5^ Accordingly, the ERAS program improved patient care and reduced direct health care costs.^5^ Our previous study also proved that a short‐stay of 3 days after THA was safe and effective for unselected patients under ERAS program.^6^ In addition, the costs‐saving benefits of reduced LOS were also verified.^6^ However, whether further decrease in LOS could influence joint function, rate of adverse events and patient satisfactory after TJA remains unknown. Surgeons may still worry about the postoperative joint function exercise after discharge, and even the occurrence of severe complication of the TJA procedure. Based on our previous study, we aimed to investigate the safety, efficiency and cost in TJA patients with LOS ≤ 2 under the short‐stay ERAS program in the current study.

Recognizing the preoperative patient characteristics related to delayed discharge after TJA is an important issue in the ERAS program. It is of vital importance to establish the optimal ERAS standards and helps in optimizing preoperative patient selection and stratifying risk of encountering prolonged LOS. If patients undergoing TJA present a predisposition for delayed discharge preoperatively, enhanced care and extended functional rehabilitation should be implemented, and the rehabilitation protocol and discharge planning for them may be changed. Also, determination of the predictors preoperatively builds the patients' expectations and may improve their satisfaction. Plenty of risk factors have been identified for prolonged LOS in western populations in the literatures, such age,^7^ sex,^8^ obesity.^9^ However, significant differences in the demographics of TJA patients exist between eastern and western populations owing to the different races and living condition. For example, Chinese TJA patients have much lower age and BMI than those reported in western literatures.^7^, ^10^ As a result, we suppose it would be crucial to identify more specific predictors of LOS to establish the optimal ERAS standards.

The aim of this current study was to: (i) investigate the safety, efficiency and cost in TJA patients with LOS ≤ 2 under the short‐stay ERAS program; and (ii) identify potential predictors facilitating further decrease in LOS, such as preoperative sleep quality, laboratory examinations and comorbidities.

## Patients and Methods

### Participants

We retrospectively collected the information of patients who underwent TJA in our single surgical team between January 2017 and June 2019. The inclusion criteria were: (i) patients undergoing primary unilateral TJA; (ii) patient with postoperative LOS ≤ 3; and (iii) patients completed 12‐month or longer follow‐up. Since the safety and efficiency of the 3‐day stay have been verified in our previous study^6^ and the target postoperative LOS in our team was 3 days, we only included patients with LOS no more than 3 days in this study. Patients with LOS ≤ 2 were set as the study group and patients with LOS = 3 were set as the control. There were no specific exclusion criteria. This study was approved by the Institutional Review Board of West China Hospital of Sichuan University (No. 201200268).

### ERAS Program

All patients received the same ERAS protocol and one single senior surgeon (Zongke Zhou) performed all TJA.

### Preoperative Preparation

Surgeons and nurses introduce the surgical plans, costs, intraoperative risks and postoperative notices to the patients. After admission, patients are encouraged to carry out joint functional exercises on the bed to increase the strength of the gluteus medius and quadriceps femoris, and improve the range of motion of joint. Preoperative functional exercises enhance the muscle memory of patients, and can make them adapt to the postoperative functional exercise more quickly. Patients are encouraged to cough, blow balloons and expand chest by raising hands to improve cardiopulmonary function. Hypoproteinemia leads to delayed wound healing and increased risk of infection after surgery. For nutritional support, patients are encouraged to have a high protein diet (at least four full eggs a day and more meat products). Patients with poor appetite can be treated with a prokinetic agent (mosapride 5mg three times a day).

### Blood Management

The strategy of blood management is composed of optimizing hematopoiesis, reducing blood loss and strict blood transfusion. Optimization of hematopoiesis mainly includes recombinant Human Erythropoietin treatment combined with chalybeate to correct preoperative anemia and enhance erythrocyte mobilization. Two grams of intravenous tranexamic acid 10 min before skin incision, and two doses 1 g of tranexamic acid at 3 and 6 h postoperatively are applied to reduce blood loss. Also, intraoperative mean arterial pressure was maintained at 60–70 mmHg to reduce intraoperative bleeding. In terms of blood transfusion, we adopt the transfusion indications in the Technical Specifications for Clinical Blood Transfusion issued by the Ministry of Health of China in 2000: hemoglobin (Hb) >100 g/L generally do not need blood transfusion; Hb < 70 g/L need blood transfusion; 70 g/L < Hb < 100 g/L is determined according to the patient's age, anemia symptoms, cardiopulmonary function and hemodynamics.

### Pain and Sleep Management

Patients receive celecoxib 200 mg every 12 h for preemptive analgesia after admission. Local infiltration analgesia with an 80 mL periarticular injection of 0.25% ropivacaine was performed. The ice compression device was applied after surgery. Oral celecoxib (200 mg, every 12 h) was administrated until 2 weeks postoperatively. Morphine (5 mg) was used intravenously as a rescue analgesic when patients experienced unbearable pain with visual analogue scale (VAS) pain score >6.

After admission, patients were given alprazolam 0.4–0.8 mg, oryzanol 30 mg, and sodium bromide 20 mL every night. Patients with symptoms of insomnia received psychological behavior intervention: improved doctor‐patient communication, ameliorative inpatient ward environment and promoted sense of security of patients. Patients with significant symptoms of depression and anxiety were given duloxetine 60 mg per day with the consultation of mental health center for medication guidance when necessary.

### Prevention of Infection and Thrombosis

Before skin excision, cefuroxime sodium 1.5 g is given to the patients (patients allergic to cefuroxime sodium receive clindamycin 600 mg). During the operation, the operative field is repeatedly rinsed and the joint cavity is soaked with povidone iodine solution. After surgery, patients are given cefuroxime sodium 1.5 g twice every 8 h to prevent infection.

After surgery, a pneumatic planta venous pump is used to prevent deep venous thrombosis in the lower extremity. After the patients return to the ward after surgery, they are immediately encouraged to perform quadriceps muscle isometric contraction, ankle dorsiflexion and toe flexion exercises. In addition, patients are given 0.2 mL low‐molecular‐weight heparin subcutaneously 6 h after surgery, and the dosage is changed to 0.4 mL once a day from the first day after surgery to discharge. After discharge, patients receive rivaroxaban 10 mg once a day or apixaban 5 mg twice a day for 10 days.

### Discharge Criteria

(i) The general status of the patient is good with normal diet; (ii) the wound is dry without bleeding and exudation; (iii) The pain is mild with a VAS score less than 3; (iv) hip flexion ≥100°, knee flexion ≥100°, hip extension between −5° and 0°, knee extension between −5° and 0°; and (v) patients can get on and off the bed, walk with a walker, go to the toilet, get on and get off the car by themselves. Patients are encouraged to go home after discharge and are followed up routinely in our hospital.

### Data Collection

#### Length of Stay

LOS was defined as the number of nights stayed in hospital after surgery, as previously reported.^11^, ^12^

#### Preoperative Sleep Quality

We used the Pittsburgh Sleep Quality Index (PSQI) score to assess the preoperative sleep quality of the patients, and lower PSQI score means higher sleep quality. PSQI ≥ 5 was defined as poor sleep quality and PSQI < 5 defined as high sleep quality according to previous literature.^13^ Other preoperative characteristics of patients, including age, gender, body mass index (BMI), ethnic group, type of surgery, Barthel Index for activities of daily living (ADL), living alone, preoperative Hb, preoperative albumin and comorbidities, were also collected from clinical records.

#### Comorbidities

Existing comorbidities diagnosed before admission and during hospitalization, including diabetes, hypertension, atherosclerosis, other cardiac diseases, cerebrovascular diseases, respiratory diseases, renal diseases, digestive diseases, osteoporosis and lumbar disease, were recorded in detail. Number of comorbidities, American Society of Anesthesiologists (ASA) grade and Charlson Comorbidity Index (CCI) score were used to assess the severity of comorbidities.

#### Joint Function

All 604 patients enrolled in the current study have been followed up for more than 12 months after surgery. Joint function was recorded at the latest follow‐up. The hip function of THA patients was assessed using Harris hip score (HHS) and the knee function of TKA patients was assessed using Hospital for Special Surgery (HSS).

#### Adverse Events

Adverse events were recorded at the latest follow‐up. Adverse events including aseptic loosening, periprosthetic joint infection, periprosthetic fracture, hip dislocation, blood transfusion, venous thrombosis, pulmonary embolism and neurovascular events, were collected in details.^14^, ^15^

#### Hospitalization Costs

Hospitalization costs for each patient, except that for prostheses, were collected from the medical record system. Chinese yuan was used as the unit of measurement.

#### Statistical Analysis

Continuous data are expressed as the mean and standard deviation (SD) and were compared using the Student's *t* test. Categorical data are expressed as the number and percentage and were analyzed using Pearson chi‐square test or Fisher exact test as appropriate. The level of significance was set at *P* value <0.05. Variables showing statistical significance in *t* test and Pearson chi‐square test were included in the multivariate logistic regression analysis to find independent preoperative predictors for LOS. Statistical analysis was performed using SPSS v22.0 (IBM, Armonk, NY, USA).

## Results

### General Results

In this study, 271 patients (44.9%) had a LOS of 2 days or less (LOS ≤ 2 group) while 333 patients (55.1%) had a LOS of 3 days (LOS = 3 group). All patients went back home after discharge without the need for rehabilitation in other hospitals.

### Univariate Analysis

The LOS ≤ 2 group had significantly younger age (56.5 ± 13.3 *vs*. 60.7 ± 13.1; t = −3.949; *P* < 0.001), larger proportion of male patients (51.7% *vs*. 38.1%; χ^2^ = 11.077; *P* = 0.001), lower BMI (23.4 ± 3.5 *vs*. 24.3 ± 3.7; t = −3.228; *P* = 0.001) and more THA (86.7% *vs*. 61.3%; χ^2^ = 48.757; *P* < 0.001) than the LOS = 3 group (Table 1). It is noteworthy to mention that LOS was correlated to preoperative sleep quality. When compared to LOS = 3 group, LOS ≤ 2 group had lower PSQI score (4.2 ± 2.7 *vs*. 4.9 ± 3.4; t = −2.601; *P* = 0.01) and more patients with high sleep quality (58.7% *vs*. 48.9%; χ^2^ = 5.674; *P* = 0.017). Preoperative albumin was significantly higher in the LOS ≤ 2 group than the LOS = 3 group (44.1 ± 4.3 *vs*. 43.2 ± 3.9; t = 2.615; *P* = 0.009). The LOS ≤ 2 group had a smaller number of comorbidities (χ^2^ = 17.‐21; *P* = 0.001) and lower grade of ASA (χ^2^ = 6.516; *p* = 0.038). With respect to one single comorbidity, the LOS = 3 group had a larger proportion of patients diagnosed with hypertension (33.3% *vs*. 23.2%; χ^2^ = 7.411; *V* = 0.006), atherosclerosis (10.2% *vs*. 5.2%; χ^2^ = 5.197; *V* = 0.023) and digestive diseases (12.0% *vs*. 5.5%; χ^2^ = 7.573; *V* = 0.006).

**TABLE 1:** Preoperative patient demographics

Demographics	LOS ≤ 2 (*n* = 271)	LOS = 3 (*n* = 333)	Statistic value	*P*
Age (years)	56.5 ± 13.3	60.7 ± 13.1	−3.949^a^	<0.001
Gender			11.077^b^	0.001
Male	140 (51.7%)	127 (38.1%)		
Female	131 (48.3%)	206 (61.9%)		
BMI (kg/m^2^)	23.4 ± 3.5	24.3 ± 3.7	−3.228^a^	0.001
Ethnic group			0.899^b^	0.343
Han	256 (94.5%)	320 (96.1%)		
Others	15 (5.5%)	13 (3.9%)		
Type of surgery			48.757^b^	<0.001
THA	235 (86.7%)	204 (61.3%)		
TKA	36 (13.3%)	129 (38.7%)		
PSQI score	4.2 ± 2.7	4.9 ± 3.4	−2.601^a^	0.01
Preoperative sleep quality			5.674^b^	0.017
High (PSQI < 5)	159 (58.7%)	163 (48.9%)		
Poor (PSQI ≥ 5)	112 (41.3%)	170 (51.1%)		
Barthel Index for ADL	89.2 ± 10.8	90.7 ± 9.4	−1.800^a^	0.072
Living alone	16 (5.9%)	12 (3.6%)	1.789^b^	0.181
Preoperative hemoglobin	134.7 ± 16.4	132.2 ± 16.7	1.828^a^	0.068
Preoperative albumin	44.1 ± 4.3	43.2 ± 3.9	2.615^a^	0.009
Diabetes	13 (4.8%)	34 (10.2%)	6.101^b^	0.14
Hypertension	63 (23.2%)	111 (33.3%)	7.411^b^	0.006
Atherosclerosis	14 (5.2%)	34 (10.2%)	5.197^b^	0.023
Other cardiac diseases	5 (1.8%)	15 (4.5%)	3.301^b^	0.069
Cerebrovascular diseases	4 (1.5%)	5 (1.5%)	0.001^b^	0.979
Respiratory diseases	7 (2.6%)	14 (4.2%)	1.170^b^	0.279
Renal diseases	15 (5.5%)	31 (9.3%)	3.025^b^	0.082
Digestive diseases	15 (5.5%)	40 (12.0%)	7.573^b^	0.006
Osteoporosis	27 (10.0%)	34 (10.2%)	0.010^b^	0.92
Lumbar disease	8 (3.0%)	6 (1.8%)	0.873^b^	0.35
Number of comorbidities			17.021^b^	0.001
0	154 (56.8%)	145 (43.5%)		
1	78 (28.8%)	97 (29.1%)		
2	28 (10.3%)	63 (18.9%)		
≥3	11 (4.1%)	28 (8.4%)		
ASA grade			6.516^b^	0.038
1	57 (21.0%)	53 (15.9%)		
2	164 (60.5%)	192 (57.7%)		
3	50 (18.5%)	88 (26.4%)		
CCI score			8.595^b^	0.072
0	59 (21.8%)	71 (21.3%)		
1	75 (27.7%)	70 (21.0%)		
2	60 (22.1%)	71 (21.3%)		
3	51 (18.8%)	65 (19.5%)		
≥4	26 (9.6%)	56 (16.8%)		

Abbreviations: ADL, activities of daily living; ASA, American Society of Anesthesiologists; BMI, body mass index; CCI, Charlson Comorbidity Index; PSQI, Pittsburgh Sleep Quality Index; THA, total hip arthroplasty; TKA, total knee arthroplasty.

^a^ Statistic value of t.

^b^ Statistic value of χ^2^.

### Multivariate Logistic Analysis Model

Factors showing significant difference in univariate analysis, including age, gender, BMI, type of surgery, PSQI score, preoperative albumin, hypertension, atherosclerosis, digestive diseases, number of comorbidities and ASA grade, were included in the multivariate logistic analysis model (Table 2). PSQI score (odds ratio [OR] = 1.084, 95% confidence interval [CI] = 1.024–1.147, *P* = 0.005), preoperative albumin (OR = 0.945, 95% CI = 0.905–0.988, *P* = 0.012), digestive diseases (OR = 1.084, 95% CI = 1.024–1.147, *P* = 0.005) and THA (OR = 0.273, 95% CI = 0.170–0.439, *P* < 0.001) were predictors of further decrease in LOS (LOS ≤ 2) in the multivariate logistic analysis model. Age, gender, BMI, hypertension, atherosclerosis, number of comorbidities and ASA grade were determined to be confounding variables.

**TABLE 2:** Multivariate logistic regression analysis identifying variables related to LOS ≤ 2

Variables	Odds ratio	95% confidence interval	*P*
Age	1.004	0.987–1.021	0.644
Female	1.416	0.981–2.044	0.063
BMI	1.042	0.991–1.097	0.109
THA	0.273	0.170–0.439	<0.001
PSQI score	1.084	1.024–1.147	0.005
Preoperative albumin	0.945	0.905–0.988	0.012
Hypertension	0.859	0.478–1.543	0.612
Atherosclerosis	2.118	0.955–4.696	0.065
Digestive diseases	2.384	1.111–5.114	0.026
Number of comorbidities			
0	1	‐	‐
1	0.963	0.571–1.625	0.888
2	1.330	0.631–2.804	0.454
≥3	0.915	0.288–2.912	0.880
ASA grade			
1	1	‐	‐
2	0.637	0.381–1.064	0.085
3	0.796	0.398–1.593	0.520

Abbreviations: ASA, American Society of Anesthesiologists; BMI, body mass index, PSQI, Pittsburgh Sleep Quality Index, THA, total hip arthroplasty.

### Preoperative Sleep Quality

**Fig. 1:**
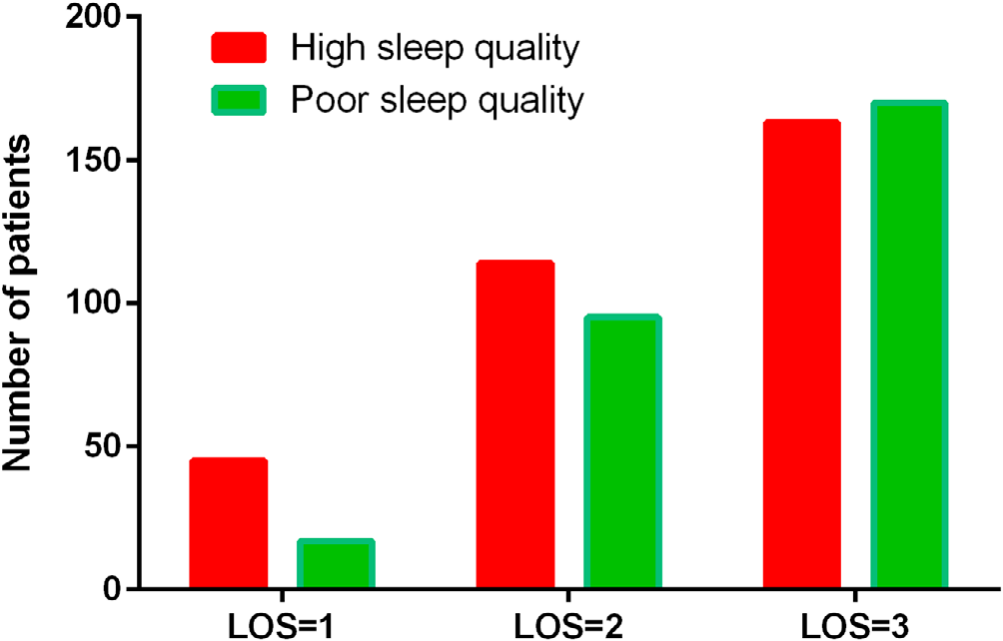
Patients with preoperative high sleep quality had significantly shorter length of stay than patients with poor sleep quality (*P* = 0.003)

Since low PSQI score (high sleep quality) was determined to be a protective factor for LOS ≤ 2, we further investigated the relationship between sleep quality and LOS, joint function, adverse events, and hospitalization costs. Patients with preoperative high sleep quality (n = 322, 53.3%) had significantly shorter LOS than patients with poor sleep quality (n = 282, 46.7%) (*P* = 0.003) (Fig. 1). However, patients with high sleep quality and patients with poor sleep quality had similar function scores, hospital costs and adverse event rates (Table 3).

**TABLE 3:** Relationship between sleep quality and joint function, adverse events, hospitalization costs

Variables	THA				TKA			
	High sleep quality (*n* = 228)	Poor sleep quality (*n* = 211)	Statistic value	*P*	High sleep quality (*n* = 94)	Poor sleep quality (*n* = 71)	Statistic value	*P*
Preoperative function	56.9 ± 9.2	56.0 ± 9.8	1.021^a^	0.308	44.4 ± 10.2	44.2 ± 9.0	0.129^a^	0.897
Postoperative function	93.7 ± 4.5	93.7 ± 4.1	−0.31^a^	0.975	91.9 ± 10.5	90.9 ± 6.1	0.695^a^	0.488
Improvement in function	36.8 ± 9.9	37.7 ± 10.4	−0.960^a^	0.338	47.5 ± 13.9	46.7 ± 10.0	0.397^a^	0.692
Hospital costs	21804.6 ± 4169.3	21843.2 ± 3892.7	−0.100^a^	0.920	22811.7 ± 3134.5	23207.2 ± 2468.3	−0.877^a^	0.382
Adverse events	2	4	‐	0.434	1	1	‐	1.000

Abbreviations: THA, total hip arthroplasty, TKA, total knee arthroplasty.

The hip function of THA patients was assessed using Harris Hip Score (HHS) and the knee function of TKA patients was assessed using Hospital for Special Surgery (HSS). PSQI ≥ 5 defined as poor sleep quality and PSQI < 5 defined as high sleep quality.

Hospital costs was expressed as Chinese yuan.

^a^ Statistic value of t.

### Joint Function, Adverse Event and Hospital Costs

In regard to the safety, efficiency and hospital cost of shorter‐stay THA patients, the preoperative HHS, postoperative HHS, cost and adverse event rate were comparable between the LOS ≤ 2 group and the LOS = 3 group (Table 4). TKA patients in the LOS ≤ 2 group showed significant better preoperative HSS (47.2 ± 7.5 *vs*. 43.6 ± 10.0; t = 0.043; *P* = 0.043) and lower hospital cost (22100.7 ± 2389.9 *vs*. 23227.8 ± 2945.9; t = −2.109; *P* = 0.037) than TKA patients in the LOS = 3 group. Nevertheless, the postoperative knee function, improvement in knee function and adverse event rate had no significant differences between two groups. All patients undergoing TJA showed significant improvement in joint function after surgery (Table 4).

**TABLE 4:** Relationship between LOS and joint function, adverse events, hospitalization costs

Variables	THA				TKA			
	LOS ≤ 2 (*n* = 235)	LOS = 3 (*n* = 204)	Statistic value	*P*	LOS ≤ 2 (*n* = 36)	LOS = 3 (*n* = 129)	Statistic value	*P*
Preoperative function	56.2 ± 10.1	56.7 ± 8.5	−0.538^a^	0.591	47.2 ± 7.5	43.6 ± 10.0	2.040^a^	0.043
Postoperative function	94.0 ± 4.5	93.3 ± 3.9	1.605^a^	0.109	91.6 ± 4.9	91.5 ± 9.7	0.111^a^	0.912
Improvement in function	37.8 ± 10.7	36.6 ± 9.5	1.179^a^	0.239	44.4 ± 7.0	47.9 ± 13.4	−1.497^a^	0.136
Hospital costs	21880.8 ± 3666.5	21756.8 ± 44.28	0.321^a^	0.752	22100.7 ± 2389.9	23227.8 ± 2945.9	−2.109^a^	0.037
Adverse event	4	2	‐	0.690	1	1	‐	1.000
Aseptic loosening	0	0	‐	‐	0	0	‐	‐
Periprosthetic joint infection	0	0	‐	‐	0	0	‐	‐
Periprosthetic fracture	0	0	‐	‐	0	0	‐	‐
Dislocation	3	1	‐	‐	‐	‐	‐	‐
Blood transfusion	0	1	‐	‐	0	1	‐	‐
Venous thrombosis	1	0	‐	‐	0	0	‐	‐
Pulmonary embolism	0	0	‐	‐	0	0	‐	‐
Neurovascular events	0	1	‐	‐	1	0	‐	‐

Abbreviations: THA, total hip arthroplasty, TKA, total knee arthroplasty.

The hip function of THA patients was assessed using Harris Hip Score (HHS) and the knee function of TKA patients was assessed using Hospital for Special Surgery (HSS).

Hospital costs was expressed as Chinese yuan.

^a^ Statistic value of t.

## Discussion

The most valuable findings of this retrospective cohort study involving the 604 TJA patients were that it was safe, efficient and economical for TJA patients to be discharged within 2 days after surgery. High preoperative sleep quality, high preoperative albumin level, free of digestive disease and THA procedure were predictors of LOS ≤ 2. This showed the significance of our study. Our results support the routinely application of 2‐day discharge after TJA in an unselected population under the ERAS program because of the safety, efficiency and cost‐saving efficacy of the 2‐day stay. We believe our results can also serve as a basis for achieving the final goal of outpatient TJA. Also, predictors for LOS found in our study may also be generalized to other institutions regardless of the exact LOS. Improving the preoperative sleep quality and preoperative albumin level through interventions of sleep management and nutritional support may facilitate early discharge in TJA patients.

### ERAS Program and LOS

The ERAS program in our study focuses on the optimization of perioperative management, mainly including preoperative preparation (patient education, preoperative functional exercises, nutritional support), blood management (optimizing hematopoiesis, reducing blood loss, strict blood transfusion), pain management (preemptive analgesia, local infiltration analgesia, rescue analgesic), sleep management, prevention of infection, prevention of thrombosis (venous pump, active exercises, anticoagulant drugs) and strict discharge criteria. Such an ERAS program facilitates the feasibility of early discharge without affecting the joint function and increasing the adverse event rate. Also, the cost‐saving effect of short‐stay under the Chinese health‐care system was confirmed by our study. The results of this study were in accordance with a previous published prospective randomized study, which also confirmed the safety and feasibility of a 2‐day discharge protocol after THA in selected patients.^16^ However, the results of this real‐world study with unselected patients have better external validity, which is a critical point for generalizing the results.

### Preoperative Sleep Quality and LOS

Sleep disorder is commonly observed in patients with osteoarthritis due to the chronic pain, reduced physical activity, poor mental health and correlated comorbidities.^17^ It has been reported that insomnia and insufficient sleep were observed in more than 75% patients with knee or hip osteoarthritis.^11^ Among patients with end‐stage osteoarthritis undergoing TJA, the symptoms of sleep disorder were more significant because of the anxiety and depression for facing the surgery.^18^ However, a limited number of studies have investigated the effect of preoperative sleep quality on postoperative outcomes and LOS in TJA patients. Our results showed that patients with high preoperative sleep quality had more rapid recovery after surgery and shorter LOS. The results can be explained by the fact that patients with low sleep quality are associated with pain catastrophizing, central sensitization and more severe pain after surgery.^19^ However, we also found that the follow‐up function and complication rate did not differ between patients with different sleep quality. This is in accordance to the findings of Kirksey *et al*. that the sleep quality was improved after 3 months in TKA patients,^20^ and it was speculated that the effect of low sleep quality on the joint function would disappear. Since interventions of sleep management may improve sleep quality in TJA patients during the perioperative period,^21^, ^22^, ^23^ we can speculate that improving preoperative sleep quality in a multimodal sleep management strategy may accelerate the postoperative recovery of the patients.

### Preoperative Albumin Level and LOS

The current study found that high preoperative albumin level were predictors of LOS ≤ 2, which was consistent with previous studies reporting low preoperative albumin level was associated with delayed discharge and increased hospital cost.^24^ Besides, preoperative hypoalbuminemia was related to various postoperative complications following TJA, including cardiac arrest, myocardial infarction, cerebrovascular accident, sepsis, pulmonary infection, impaired renal function, wound infection, periprosthetic joint infection and even mortality.^25^, ^26^ Albumin is the most reliable and commonly used indicator to reflect the nutritional status of the patients, and our results confirm the role of preoperative albumin level in predicting LOS under a short‐stay ERAS program. Human albumin solution is often used to correct hypoalbuminemia during the perioperative period, but a study that enrolled over 1 million TJA patients in the United States showed that the use of human albumin solution increased the risk of acute renal failure, thromboembolic complications, cardiac complications, pulmonary complications and intensive care unit admission rates.^27^ Surgeons should consider optimizing the nutritional status and albumin level of TJA patients prior to surgery. Nutritional support through wide‐ranging and specific nutritional substrates (high protein) supplements plays an important role in optimizing the nutritional status of the patients and stimulate the faster recovery after TJA.^28^

### Comorbidities and LOS

Our results showed that single comorbidity, number of comorbidities and severity of comorbidities all had limited influence on LOS (except digestive diseases). The results were quite different from the findings of many previous studies, which showed comorbidities were crucial predictors for prolonged LOS.^29^, ^30^, ^31^ We believe the perioperative management of comorbidities in our team can minimize their influence on LOS. First, patients were strictly screened for comorbidities and assessed to determine if they were eligible for the TJA surgery. Once the patients were confirmed to accept TJA, aggressive and accurate management of the comorbidities was immediately undertaken to optimize patient status. Also, a minimally invasive surgery technique was applied to decrease surgical stress and avoid fluctuation of the comorbidities. The reason why digestive diseases had a weak influence on LOS might be that the digestive diseases have an adverse impact on the nutritional status, mental state and ultimately affect recovery.

## Limitations

Our study has several limitations. First, recent studies in the western countries have been investigating the feasibility of TJA as an outpatient procedure, but the safety of outpatient TJA in unselected patients still concerns the surgeons at present. Some studies have demonstrated that outpatient TJA has similar outcomes with inpatient TJA.^32^, ^33^, ^34^ However, most of these studies had a selected population for patients who underwent outpatient TJA. Some research even found outpatient TJA had a higher risk of perioperative and postoperative complications.^35^, ^36^ On the other hand, outpatient TJA may be not applicable in the Chinese population at present because of the different perspective on medicine of patients, medical care policy and socioeconomic issues. As a result, the significance of our study is clear. We proved the safety, efficiency and cost‐saving efficacy of 2‐day stay in Chinese unselected patients, and we believe our results can serve as a basis for achieving the final goal of a 1‐day stay. Also, predictors for LOS found in our study may be also generalized to other institution regardless of the exact LOS (e.g., high preoperative sleep quality predicts shorter LOS). Second, measuring LOS by hours rather than days is more accurate and may provide more information about discharge. However, discussing LOS by days favors the classification of patients to delayed discharge or on‐time discharge, and helps find the relationship between LOS predictors and LOS. To our knowledge, most previous publications reported LOS by days.^12^, ^37^, ^38^ Third, the sample size of this study was relatively small when compared to studies from national registers. However, all patients from one single medical team guaranteed the ERAS protocol in the study was consistent. A total of 604 TJA patients also gave our study enough power to investigate our outcomes.

## Conclusion

In conclusion, it is safe, feasible and economical for unselected TJA patients to discharge within 2 days after surgery. Patients with high sleep quality, with high preoperative albumin, without digestive diseases and undergoing THA are more likely to discharge within 2 days after surgery. Routine application of 2‐day discharge after TJA under ERAS program can be recommended. Improving the preoperative sleep quality and preoperative albumin level through critical sleep management and nutritional support may facilitate early discharge in TJA patients.

## Authors’ Contribution

Zichuan Ding: Conceptualization, Methodology and Writing; Jinlong Li: Data collection; Bing Xu: Data collection; Cao Jian: Visualization; Hao Li: Software; Zongke Zhou: Conceptualization, Supervision and Funding acquisition.
